# Relationships between Hamstrings-to-Quadriceps Ratio and Variables Describing Countermovement and Drop Jumps

**DOI:** 10.1155/2019/4505481

**Published:** 2019-06-02

**Authors:** Artur Struzik, Bogdan Pietraszewski

**Affiliations:** ^1^Department of Team Sport Games, University School of Physical Education, Wrocław, Poland; ^2^Department of Biomechanics, University School of Physical Education, Wrocław, Poland

## Abstract

The impact of the hamstrings-to-quadriceps ratio on sport movement performance has not been sufficiently described. However, it seems that in movements involving eccentric-concentric muscular contractions, a higher hamstrings-to-quadriceps ratio should have a positive impact on human movement performance. The present study is aimed at identifying relationships between the hamstrings-to-quadriceps ratio and variables describing countermovement and drop jumps. The study was carried out in a group of 14 female soccer players. The tests were conducted using a Kistler force plate, an SG electrogoniometer, and the Biodex System 4 Pro dynamometer. Each player performed three countermovement jumps (CMJ) and three drop jumps (DJ) from heights of 15, 30, 45, and 60 cm. The peak torques of knee extensors and flexors were measured in isometric conditions and in isokinetic conditions at angular velocities of 30^o^/s, 60^o^/s, 90^o^/s, and 120^o^/s. Statistically significant relationships were found between the variables that describe CMJ, DJ 15, DJ 30, and hamstrings-to-quadriceps ratio at some, though not all, of the angular velocities measured. No significant relationships were found between the hamstrings-to-quadriceps ratio and variables that describe DJ 45 and DJ 60. The heights of CMJ, DJ 15, and DJ 30 were increased with higher hamstrings-to-quadriceps ratios. Analogous relationships were found between the hamstrings-to-quadriceps ratio and relative mechanical power during the take-off phase of the CMJ. Significant relationships between the hamstrings-to-quadriceps ratio and variables that describe vertical jump are likely to be observed if adequate angular velocity is used in the measurement of muscle torque.

## 1. Introduction

The commonly used conventional hamstrings-to-quadriceps (H/Q) ratio represents the ratio of concentric hamstring peak torque during lower limb flexion to concentric quadricep peak torque during lower limb extension [1]. The effect of the peak torque ratio of knee flexors and extensors on the risk of injury in the lower limbs has been extensively discussed in the literature [2–6]. However, the relationships between the value of the H/Q ratio and sport movement performance have not been sufficiently explored to date. It is generally accepted that a value of the flexor-extensor ratio above 0.6 (for measurements performed under isokinetic conditions) might be effective at preventing hamstring strain and anterior cruciate ligament (ACL) injury risk potential [2–6]. However, the question arises as to whether relatively high levels of muscle torque in knee flexors in comparison with extensors might positively affect sports movement performance.

Maximum values of isometric muscle torque in extensors and flexors of the knee joint have been demonstrated to not reliably predict the level of jumping ability [7, 8]. Kubo et al. [9] reported that isometric training changes the stiffness of the tendon-aponeurosis complex in knee extensors and negatively impacts prestretch during the stretch-shortening cycle (SSC). It is likely that muscular activation is different between static and dynamic actions [8]. However, Trzaskoma [10] found positive relationships between maximal power output developed during countermovement jumps (CMJ) and the value of the H/Q ratio calculated based on measurements of muscle torques performed under isometric conditions.

Positive relationships between the level of muscle torque and variables that describe vertical jump are, however, significant if the measurement of muscle torque is performed in isokinetic rather than isometric conditions [11–15]. Martel et al. [16] reported that plyometric training might lead to the improvement of both CMJ height and the values of muscle torque in knee flexors and extensors measured at angular velocities of 60^o^/s and 180^o^/s. Furthermore, Wilkerson et al. [17] reported that plyometric training results in an increase in the H/Q ratio for measurements taken at an angular velocity of 60^o^/s.

CMJ, drop jump (DJ), and the movements performed during the measurement of knee muscle torque under isokinetic conditions (especially with high measurement angular velocity) all involve the SSC. The alternating eccentric-concentric muscle work leads to the accumulation of potential elastic energy, consequently allowing for more work to be performed in the concentric phase [18]. It seems that during movements with eccentric-concentric muscle work, higher values of the H/Q ratio should have a positive effect on human movement performance. Therefore, the aim of the study is to identify the relationships between the value of the H/Q ratio and variables that describe CMJ and DJ vertical jumps. Due to the alternate eccentric-concentric muscle work during the countermovement (amortization) and take-off phases of the vertical jump, it seems that the conventional H/Q ratio is more suitable for describing human movement performance during SSC compared to the functional H/Q ratio, which is a measure of the isokinetic eccentric peak torque of the hamstrings relative to the isokinetic concentric peak torque of the quadriceps during leg extension at equivalent angular velocities [19].

## 2. Materials and Methods

The study population consisted of 14 female soccer players from the Polish Women's Extra League. The study group was characterized by the following mean parameters (±SD): body height—166.6 ± 5.8 cm, body mass—59.3 ± 6 kg, and age—20.6 ± 4.1 years. Training experience was 8.8 ± 3.9 years. The tests were carried out in the Biomechanical Analysis Laboratory (with PN-EN ISO 9001:2009 certification). Prior to the measurements, the participants were familiarized with the purpose of the study and gave written consent for participating in the experiment. Before the test, the subjects were informed of the activities they were supposed to perform and were motivated to properly perform the task. The research project was approved by the Senate's Research Bioethics Commission, and the procedure complied with the Declaration of Helsinki regarding human experimentation.

Prior to the measurements, a 10-minute-long warm-up was administered, which included jogging, a series of hops, and rehearsing the drop jumps.

**Figure 1:**
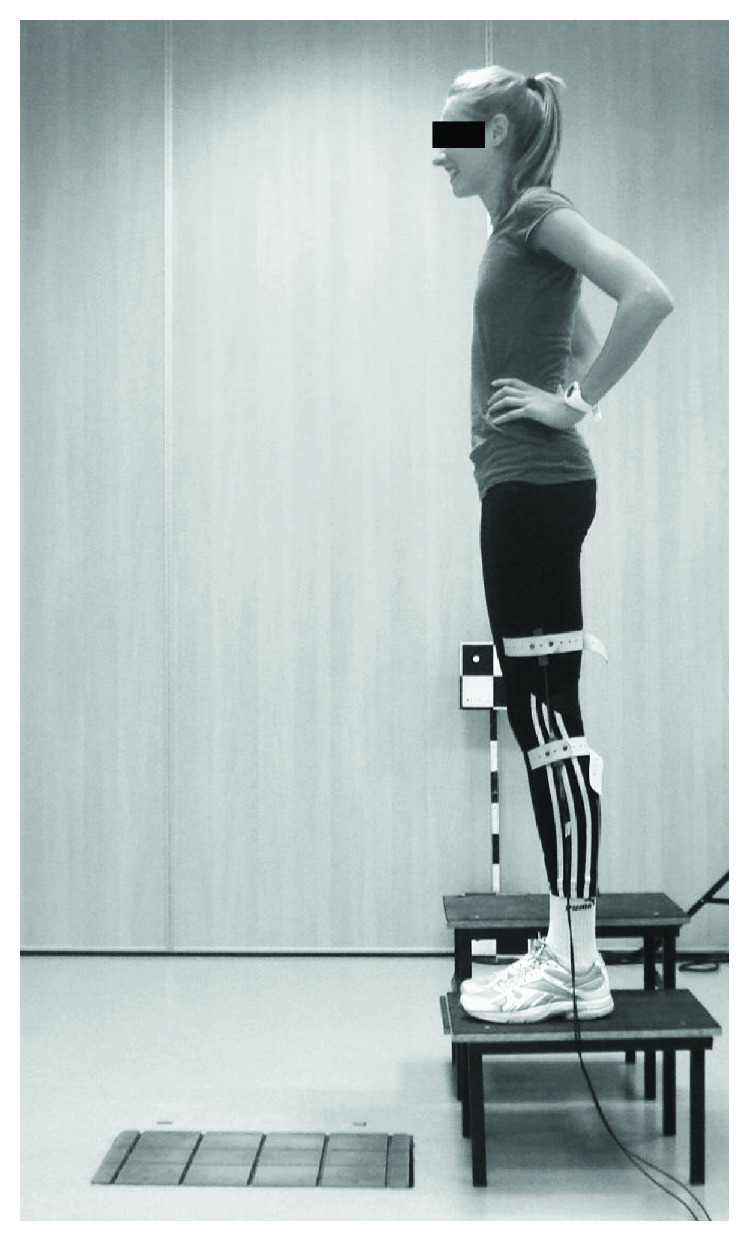
Drop jump (DJ) laboratory testing set-up.

The participants were asked to perform CMJ (countermovement jump, which is a vertical jump with an arm swing) and DJ (drop jump, which is a vertical jump performed immediately after landing) from heights of 15, 30, 45, and 60 cm (Figure 1). Each jump was repeated three times, and further analysis was based on the highest jump for each type (and for each participant). Ground reaction forces were recorded by means of the 9281B13 Kistler force plate (Switzerland) with a signal sampling frequency of 250 Hz. The SG Biometrics electrogoniometer in the knee joint was used for recording temporary changes in the angle in this joint. A comparison of the profile of angle changes in the knee joint with the vertical component of ground reaction forces was used to determine the ground contact time (*t*_c_), amortization time (*t*_a_), and take-off time (*t*_p_) [7, 20]. Jump height was calculated based on the flight phase duration. Relative mechanical power (*P*_rel_) in the take-off phase was calculated based on the equation contained in a study by Pietraszewski and Rutkowska-Kucharska [20]:

(1) $$\begin{array}{c} P_{\text{rel}}=\frac{g∙h_{\text{j}}}{t_{\text{p}}}, \end{array}$$

where *h*_j_ denotes the jump height, *t*_p_ the take-off time, and *g* the acceleration due to gravity. Power calculated using this equation relates to the vertical displacement of the centre of mass of a jumper from the moment of take-off to the maximal location during the flight phase.

**Figure 2:**
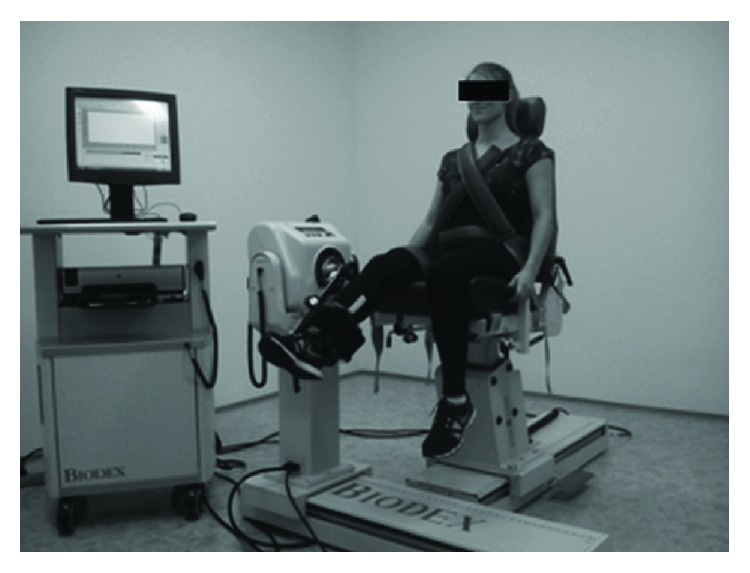
Test stand for the measurement of muscle torque.

Peak muscle torques for extensors and flexors of the knee joint were also measured under isometric conditions (for the angles of 75^o^ and 30^o^, respectively) and under isokinetic conditions (at angular velocities *ω* 30^o^/s, 60^o^/s, 90^o^/s, and 120^o^/s in the joint analysed) for each subject. 0^o^ at the knee joint was considered to be a full extension. The measurements were performed separately for the right and left lower limbs. Biodex System 4 Pro was used for the measurements (Figure 2). The measurements under isokinetic conditions (with eccentric-concentric muscle work) were performed with a range of motion in the knee joint of 90^o^ (from 90^o^ to 0^o^). The hamstrings-to-quadriceps ratio (H/Q ratio) was calculated using the following equation:

(2) $$\begin{array}{c} \text{H}/\text{Q ratio}=\frac{F_{l}+F_{r}}{E_{l}+E_{r}}∙100\%, \end{array}$$

where (*F*_*l*_ + *F*_*r*_) denotes the sum of the values of peak torque developed in the knee joint flexors in the left and right lower limbs, whereas (*E*_*l*_ + *E*_*r*_) is the analogous sum for the extensors [10, 21]. This calculation method was used intentionally because all types of jumps used in the study were performed with both lower limbs. Therefore, we used the calculation of an index which applies to both lower limbs at the same time.

Furthermore, the research group was divided into two subgroups based on the values obtained for the H/Q ratio. The subgroup “good” were people (*n* = 7) with higher H/Q ratios compared to the second subgroup (“poor”). The above division was aimed at comparing jump variables between the subgroups.

Pearson's *r* coefficient and Student's *t*-test were used to examine the relationships between the selected variables due to the normality of the distribution of the variables. Additionally, the *t*-test for independent groups was used to investigate the differences between the “good” and “poor” subgroups. The level of significance was set at *α* = 0.05.

## 3. Results

Table 1 contains the mean values (±SD) of the variables that describe the CMJ and DJ. In the group of female soccer players, the mean value of the H/Q ratio for the torques measured under isometric conditions was 42 ± 3.6%, whereas for the measurements performed under isokinetic conditions, the mean values were 48.8 ± 7.4% (for *ω* = 30^o^/s), 54.1 ± 7.4% (for *ω* = 60^o^/s), 55.2 ± 7.2% (for *ω* = 90^o^/s), and 55.1 ± 8.3% (for *ω* = 120^o^/s).

Table 2 contains the values of the correlation coefficients between the variables that describe CMJ and DJ and the values of the H/Q ratio. Statistically significant positive relationships (*p* < 0.05) were found between the H/Q ratio and CMJ height (for isometric and for *ω* = 30^o^/s, 60^o^/s, 90^o^/s, and 120^o^/s), DJ 15 (for *ω* = 60^o^/s, 90^o^/s, and 120^o^/s), and DJ 30 (for *ω* = 120^o^/s). The H/Q ratio also showed a statistically significant relationship (*p* < 0.05) with relative mechanical power during the take-off phase of the CMJ (for *ω* = 60^o^/s, 90^o^/s, and 120^o^/s). No statistically significant relationships were found between the H/Q ratio and the variables that describe DJ 45 and DJ 60. Statistically significant relationships (*p* < 0.05) were observed between the H/Q ratio and the duration of amortization, take-off, and contact phases for the DJ 30 only.

No statistically significant differences in jump variables (for all types of jumps) were found between the “good” and “poor” subgroups, despite the fact that the “good” group was characterized on average by about 10% higher H/Q ratios.

## 4. Discussion

Soccer is a sport involving a predominance of explosive movements that require a substantial level of speed-strength abilities. Although female soccer players do not jump a lot during a match, they perform other movements that utilize the SSC, such as strikes, turns, changes in direction, and cuts [22]. Therefore, the jumping ability of soccer players could still be considered crucial for performance [23, 24]. The adequate strength of the hamstring muscles not only ensures the effective braking of the shank when striking a ball but also plays an important role in other movements, including rapid acceleration/deceleration, cutting, or side-stepping manoeuvres [2, 12, 25, 26]. Fousekis et al. [27] found a slight increase in the H/Q ratio with greater amounts of professional training in soccer players. Cometti et al. [28] demonstrated that sports skill level in soccer players increases with higher H/Q ratios. A similar tendency was not observed regarding the CMJ height [28]. This may be the reason why no significant differences in jump variables were observed between the “good” and “poor” subgroups in this study. Furthermore, Daneshjoo et al. [3] suggested that the physical performance and movement pattern experienced when playing soccer should not have a negative effect on the H/Q ratio in the knee joint. The values of the H/Q ratio were similar across different field positions for elite collegiate American football players [29].

It is difficult to draw unequivocal conclusions concerning the relationships between the H/Q ratio and the variables describing the vertical jumps studied based on the values of the correlation coefficients in Table 2. Statistically significant and positive relationships between the H/Q ratio and the height of CMJ, DJ 15, and DJ 30 allow for the assumption that the relatively high level of torque in the knee joint flexors compared to that in the extensors should positively affect the vertical jump height. However, the above relationships for the DJ 45 and DJ 60 were not statistically significant, although the heights obtained for individual types of jumps were nearly identical (Table 1). It should also be noted that high H/Q ratios may exist in the presence of relative weakness in both the hamstring and quadriceps muscle groups [17], which can also explain the lack of the above relationships. This may be another reason why no significant differences in jump variables were observed between the “good” and “poor” subgroups in this study. The female soccer players examined in the study showed mean values below 0.6 for the H/Q ratio (for isometric and for *ω* = 30^o^/s, 60^o^/s, 90^o^/s, and 120^o^/s), which is regarded as a lower limit necessary for the prevention of lower limb injuries [2–6, 30]. Lehance et al. [12], despite determining statistically significant positive relationships between peak torques of joint flexors and extensors (measured under isokinetic conditions) and squat jump (SJ) height, did not find similar relationships between the H/Q ratio (conventional and functional) and SJ height. The SJ, however, does not have an SSC pattern. González-Ravé et al. [31] did not find relationships between knee flexion and extension torque and vertical jump (CMJ and SJ) variables.

The statistically significant positive relationship between the CMJ height and H/Q ratio calculated based on the measurements of torques carried out under isometric conditions seems surprising. The relationships between the variables of the vertical jump and isometric torques in the area of the knee joint are insignificant and occur only in specific research groups [7, 8]. The explanation for the widespread occurrence of this phenomenon is likely to be found in the differing levels of muscular activation between static and dynamic tasks [8].

The H/Q ratio also showed a statistically significant relationship with relative mechanical power during the take-off phase of the CMJ. The presence of analogous similar relationships has been reported previously by other authors [10, 11, 13]. The most statistically significant relationships between the H/Q ratio and variables of vertical jumps were observed for the CMJ. Therefore, it can be presumed that an adequate ratio of muscle torque between the agonist and antagonist muscle groups for this type of jump is the most critical for performance among all the types of jump analysed. Tansel et al. [32] reported that training oriented at the development of hamstring strength should positively affect the eccentric knee extension torque and the CMJ height.

Statistically significant positive relationships were also observed between the times of contact, amortization, and take-off during the DJ 30 and the values of the H/Q ratio. We found no other studies that have described this type of relationship. However, the positive value of the correlation coefficients might be surprising since it suggests that the increase in the level of torque in knee joint flexors compared to extensors also causes an increase in the times of contact, amortization, and take-off. It should be expected that the flexors of the knee joint help to decelerate the fall during the DJ, which would lead to a faster transition into the take-off phase. The above relationships might suggest the insignificant contribution of the knee joint flexors in the DJ. Therefore, a relatively greater contribution in the phases of amortization and take-off would be expected in the knee extensors [33]. DeStaso et al. [34] demonstrated that knee extension concentric relative peak torque is the most significant predictor of DJ height (from 50 cm platform).

Iossifidou et al. [11] suggest that there are important differences in muscle activation and knee joint power development that must be taken into consideration when torque isokinetic measurements are used to predict jumping performance. Based on the obtained relationships between the H/Q ratio and the variables of the vertical jumps, it seems that the greater the height from which the jump is performed, the greater is the angular velocity needed when measuring knee joint muscle torque for the prediction of jumping ability (jump height) [35, 36]. The presence of relationships between the H/Q ratio and the variables that describe the vertical jump is likely to be observed if adequate angular velocity is used for measuring muscle torque (particularly for the DJ). The limitations of this study may be a small size (one entire team; but because of injuries and transfers to other clubs, only 14 players out of the initial 20 participated in the full set of tests) and type (female soccer players; therefore, the investigated relationships in men or athletes from other sports do not have to be consistent with those presented in this study) of the studied group.

## 5. Conclusions

1. In the group of female soccer players, the height of the DJ 15 was greater for larger H/Q ratios (determined based on torque developed under isokinetic conditions for the angular velocities of 60°/s, 90°/s, and 120°/s). Similar relationships were found between the DJ 30 height and the H/Q ratio with muscle torque developed at an angular velocity of 120°/s
2. The height of the CMJ was greater for larger H/Q ratios based on torque measured under isometric and isokinetic conditions (for the angular velocities of 30°/s, 60°/s, 90°/s, and 120°/s).
3. The relative mechanical power during the take-off phase of the CMJ was also greater for larger H/Q ratios, as determined with torque measured under isokinetic conditions (for the angular velocities of 60°/s, 90°/s, and 120°/s).
4. We found no significant relationships between the H/Q ratio and the variables describing the DJ 45 and DJ 60.

## Figures and Tables

**Table 1:** Mean values (±SD) of jump height (*h*_j_), relative mechanical power during the take-off phase (*P*_rel_), contact time (*t*_c_), amortization time (*t*_a_), and take-off time (*t*_p_) obtained during CMJ and DJ.

	CMJ	DJ 15	DJ 30	DJ 45	DJ 60
*h*_j_ (m)	0.29 ± 0.04	0.28 ± 0.04	0.28 ± 0.05	0.28 ± 0.05	0.28 ± 0.06
*P*_rel_ (W/kg)	10.9 ± 2.1	17.0 ± 3.3	16.8 ± 3.9	17.9 ± 5.3	17.1 ± 5.7
*t*_c_ (s)	n/a	0.32 ± 0.05	0.32 ± 0.08	0.3 ± 0.06	0.31 ± 0.05
*t*_a_ (s)	n/a	0.15 ± 0.03	0.16 ± 0.04	0.14 ± 0.03	0.14 ± 0.04
*t*_p_ (s)	0.26 ± 0.04	0.16 ± 0.03	0.16 ± 0.04	0.15 ± 0.03	0.17 ± 0.03

**Table 2:** Values of the correlation coefficients between the height of the vertical jump (*h*_j_), relative mechanical power during the take-off phase (*P*_rel_), contact time (*t*_c_), amortization time (*t*_a_), take-off time (*t*_p_), and values of the H/Q ratio calculated based on measurements under isometric and isokinetic conditions.

	H/Q ratio				
	Isometric	30^o^/s	60^o^/s	90^o^/s	120^o^/s
CMJ					
*h*_j_	0.46^∗^	0.49^∗^	0.41^∗^	0.44^∗^	0.62^∗^
*P*_rel_	0.13	0.24	0.47^∗^	0.48^∗^	0.42^∗^
*t*_p_	0.12	−0.05	−0.3	−0.3	−0.09
DJ 15					
*h*_j_	0.37	0.37	0.44^∗^	0.47^∗^	0.59^∗^
*P*_rel_	0.19	0.13	0.31	0.3	0.31
*t*_c_	0.15	0.4	0.21	0.16	0.35
*t*_a_	−0.01	0.29	0.22	0.08	0.17
*t*_p_	0.19	0.35	0.07	0.16	0.25
DJ 30					
*h*_*j*_	0.22	0.23	0.28	0.3	0.48^∗^
*P*_rel_	−0.01	−0.25	0.01	−0.09	0.08
*t*_c_	0.31	0.67^∗^	0.41^∗^	0.45^∗^	0.51^∗^
*t*_a_	0.14	0.57^∗^	0.34	0.32	0.48^∗^
*t*_p_	0.37	0.6^∗^	0.32	0.37	0.45^∗^
DJ 45					
*h*_j_	0.06	0.14	0.37	0.33	0.4
*P*_rel_	−0.2	−0.28	0.07	−0.03	−0.15
*t*_c_	0.11	0.26	0.08	0.02	0.28
*t*_a_	−0.04	0.11	−0.1	−0.19	0.13
*t*_p_	0.14	0.33	0.12	0.17	0.23
DJ 60					
*h*_j_	0.01	0.02	0.21	0.2	0.26
*P*_rel_	0.02	−0.26	−0.01	−0.04	0.14
*t*_c_	−0.06	0.38	0.15	0.23	0.09
*t*_a_	0.17	0.08	0.06	0.12	0.34
*t*_p_	−0.18	0.36	0.13	0.17	−0.06

^∗^Statistically significant for *p* < 0.05.

## Data Availability

The data used to support the findings of this study are available from the corresponding author upon request.
